# Editorial: Resistance training for the oncology patient

**DOI:** 10.3389/fonc.2026.1791585

**Published:** 2026-02-23

**Authors:** Arkaitz Castañeda-Babarro, Aitor Martinez Aguirre-Betolaza

**Affiliations:** 1 Health, Physical Activity, and Sports Science Laboratory, Department of Physical Activity and Sports, Faculty of Education and Sport, University of Deusto, Bizkaia, Spain; 2 Department of Physical Activity and Sports Sciences, Faculty of Health Sciences, Euneiz University, Vitoria-Gasteiz, Spain

**Keywords:** cancer, muscle, Quality of Life, sarcopenia, strength

Cancer remains one of the leading causes of morbidity and mortality worldwide, with a steadily increasing incidence driven by population aging, lifestyle factors, and improved diagnostic capabilities (1). Advances in early detection and oncological treatments have substantially improved survival rates, transforming cancer into a chronic condition for millions of individuals. Consequently, the focus of cancer care has progressively shifted from survival alone toward long-term health, functional capacity, and quality of life across the cancer continuum. This paradigm shift has highlighted the importance of supportive care strategies that mitigate treatment-related side effects and promote physical, psychological, and social well-being in oncology patients.

Beyond pharmacological and surgical interventions, a growing body of evidence supports the role of lifestyle-related strategies, particularly physical exercise, as a safe and effective adjunct therapy in oncology. Exercise interventions have demonstrated benefits in reducing cancer-related fatigue, preserving physical function, improving quality of life, and attenuating psychological distress during and after treatment (2, 3). Moreover, observational studies indicate that higher levels of physical activity after cancer diagnosis are associated with reduced cancer-specific and all-cause mortality across several tumor types (4). These findings have led major scientific and clinical organizations to recommend the systematic integration of exercise into standard cancer care, emphasizing that oncology patients should avoid inactivity whenever medically feasible.

Among the different exercise modalities, resistance training has emerged as a particularly relevant intervention for oncology patients. Cancer and its treatments are commonly associated with sarcopenia, muscle weakness, and functional decline, which negatively affect autonomy, treatment tolerance, and long-term prognosis (5). Resistance training directly targets these sequelae by stimulating neuromuscular adaptations, preserving or increasing lean mass, and improving muscular strength and functional performance. Beyond musculoskeletal benefits, resistance exercise has been linked to favorable effects on systemic inflammation, metabolic health, immune modulation, and psychosocial outcomes, positioning it as a key component of comprehensive exercise prescriptions in oncology (6).

Over the last two decades, randomized controlled trials and systematic reviews have consistently shown that resistance training is safe and feasible across a wide range of cancer types and treatment phases. Evidence indicates that resistance exercise, performed alone or in combination with aerobic training, improves muscle strength, physical function, and health-related quality of life in cancer patients and survivors (2, 7). Recent high-quality evidence also suggests that resistance training may exert beneficial effects on cancer-related fatigue, particularly when initiated during anticancer therapy, although certainty varies depending on timing, intensity, and cancer type (8). Importantly, adverse events related to resistance training are rare and generally mild when interventions are appropriately supervised and individualized.

Despite this growing body of evidence, important gaps remain. Existing systematic reviews highlight substantial heterogeneity in resistance training protocols, including variability in intensity, volume, frequency, exercise selection, supervision, and progression strategies (7). Furthermore, much of the available evidence is derived from studies in breast and prostate cancer, limiting generalizability to other tumor entities and treatment contexts. Critical questions persist regarding the optimal resistance training dose for specific cancers, the most effective timing relative to surgery, chemotherapy, radiotherapy, or immunotherapy, and the methodological approaches best suited to individual patient characteristics such as age, baseline fitness, treatment-related toxicities, and comorbidities (2, 8).

The Research Topic *Resistance Training for The Oncology Patient* was developed to contribute to this evolving field by bringing together original research and reviews that address resistance training across diverse oncology populations, clinical settings, and delivery models. Collectively, the articles included in this Research Topic reflect the increasing clinical relevance and methodological diversity of contemporary exercise oncology research.

One contribution provides a comprehensive systematic review examining the effects of resistance exercise, alone or combined with other exercise modalities, on physical fitness, quality of life, and fatigue in cancer patients. By synthesizing evidence from multiple randomized and clinical trials, this review confirms consistent improvements in muscular strength and quality of life while reporting mixed findings for fatigue outcomes, reinforcing the need for further high-quality research focusing on intervention characteristics and outcome specificity.

Another study included in this Research Topic evaluates quality of life, fatigue, and muscle strength in women undergoing chemotherapy or hormonal therapy for breast cancer. By comparing treated patients with healthy controls, the authors demonstrate the extent of treatment-related impairments and emphasize the clinical importance of preserving muscular strength during active treatment, further supporting the integration of resistance training as a supportive intervention during therapy.

The Research Topic also includes original research exploring individualized home-based exercise programs with resistance-oriented components in patients with head and neck cancer. This study demonstrates that tailored, low- to moderate-intensity home training is safe and feasible and can lead to meaningful improvements in physical functioning and selected quality-of-life domains, addressing common barriers to supervised exercise participation in this population.

In addition, innovative delivery models are addressed through a feasibility study investigating telemedicine-based adapted physical activity programs, including resistance exercises, in pediatric oncology patients. The findings highlight the potential of remote and hybrid interventions to improve access to structured exercise programs and support physical and psychosocial well-being during active oncological care.

Finally, a narrative review on personalized exercise programs in oncology situates resistance training within a broader framework of individualized supportive care. This contribution underscores the need to move beyond one-size-fits-all prescriptions and highlights emerging strategies to tailor resistance training according to tumor type, treatment modality, and patient-specific characteristics, thereby enhancing safety, adherence, and clinical effectiveness.

Together, the articles in this Research Topic advance current knowledge on resistance training as a fundamental component of supportive oncology care. They reinforce existing evidence while identifying key gaps that must be addressed to optimize exercise prescription for oncology patients. Future research should prioritize mechanistic studies, dose–response trials, and long-term investigations across diverse cancer populations. Interdisciplinary collaboration between oncology, exercise science, and rehabilitation professionals will be essential to translate this growing evidence base into routine clinical practice.

In conclusion, resistance training represents a safe, feasible, and effective intervention to counteract many of the physical and psychosocial challenges faced by oncology patients. By integrating diverse perspectives and methodological approaches, this Research Topic contributes to the continued development of exercise oncology and supports the implementation of resistance training as a core element of comprehensive cancer care.
